# DNA damage induces Yap5-dependent transcription of *ECO1/CTF7* in *Saccharomyces cerevisiae*

**DOI:** 10.1371/journal.pone.0242968

**Published:** 2020-12-29

**Authors:** Michael G. Mfarej, Robert V. Skibbens

**Affiliations:** Department of Biological Sciences, Lehigh University, Bethlehem, Pennsylvania, United States of America; Universita degli Studi di Milano, ITALY

## Abstract

Yeast Eco1 (ESCO2 in humans) acetyltransferase converts chromatin-bound cohesins to a DNA tethering state, thereby establishing sister chromatid cohesion. Eco1 establishes cohesion during DNA replication, after which Eco1 is targeted for degradation by SCF E3 ubiquitin ligase. SCF E3 ligase, and sequential phosphorylations that promote Eco1 ubiquitination and degradation, remain active throughout the M phase. In this way, Eco1 protein levels are high during S phase, but remain low throughout the remaining cell cycle. In response to DNA damage during M phase, however, Eco1 activity increases—providing for a new wave of cohesion establishment (termed Damage-Induced Cohesion, or DIC) which is critical for efficient DNA repair. To date, little evidence exists as to the mechanism through which Eco1 activity increases during M phase in response to DNA damage. Possibilities include that either the kinases or E3 ligase, that target Eco1 for degradation, are inhibited in response to DNA damage. Our results reveal instead that the degradation machinery remains fully active during M phase, despite the presence of DNA damage. In testing alternate models through which Eco1 activity increases in response to DNA damage, the results reveal that DNA damage induces new transcription of *ECO1* and at a rate that exceeds the rate of Eco1 turnover, providing for rapid accumulation of Eco1 protein. We further show that DNA damage induction of *ECO1* transcription is in part regulated by Yap5—a stress-induced transcription factor. Given the role for mutated *ESCO2* (homolog of *ECO1*) in human birth defects, this study highlights the complex nature through which mutation of *ESCO2*, and defects in *ESCO2* regulation, may promote developmental abnormalities and contribute to various diseases including cancer.

## Introduction

In order for a cell to divide, the cell division cycle must accomplish two major feats with high fidelity. First, the genetic material must be accurately replicated during S phase. Second, the cell must segregate the duplicated genetic material to each daughter cell during M phase. To identify over time the products of chromosome duplication, the cell tethers together sister chromatids during S phase. Sister chromatid tethers, or cohesins, are comprised of a group of conserved proteins (Smc1, Smc3, Mcd1/Scc1/Rad21) and auxiliary factors (Pds5 and Scc3/Irr1) that maintain sister identity until anaphase onset [1–4]. Cohesion establishment during S phase requires an additional essential factor, Eco1/Ctf7 (herein Eco1), that is part of the GNAT-family of N-acetyltransferases [5–7]. Eco1 acetylates the Smc3 subunit of cohesin, converting chromatin-bound cohesins to a tether-competent state [7–10]. At the cellular level, mutations in *ECO1* result in aneuploidy, reduced chromatin condensation reactions, and increased sensitivity to DNA damage [5,11–14]. In humans, mutations in *ECO1* orthologs (termed *ESCO1/EFO1* and *ESCO2/EFO2*) collectively correlate with numerous forms of cancer, including melanoma and prostate cancer, and a severe developmental abnormality called Roberts Syndrome (RBS) [15–20].

Early cell cycle mapping studies revealed that Eco1 function is required only during S phase and linked Eco1 function to the DNA replication processivity factor PCNA [5]. This link between Eco1, cohesion establishment, and DNA replication factors is now well established [3,5,6,8–10,21–31]. The mechanism through which Eco1 function is limited to S phase, however, was only recently elucidated. At the end of S phase, Eco1 is phosphorylated (S99) by the cell cycle regulator Cdk1. Eco1^S99-P^ becomes a target for second-site phosphorylation (S98) by Dbf4-Cdc7 (DDK) kinase complex, a regulator of DNA replication initiation [32–35]. Eco1^S98-P, S99-P^ becomes a target for third-site phosphorylation by the GSK-3 signaling kinase Mck1, which in turn phosphorylates Eco1 at S94. Eco1^S98-P, S99-P, S94-P^ is then ubiquitinated by SCF and subsequently degraded following DNA replication [34,36]. Notably, XEco2 (Xenopous) and ESCO2 also are degraded following DNA replication [17,37,38], revealing that limiting Eco1 function to S phase is highly conserved through evolution.

Although sister chromatid tethering is typically restricted to S phase, a special form of cohesion is established during M phase in response to a double strand break (DSB)—an activity termed damage-induced cohesion (DIC) [12,13,39,40]. Beyond occurring in different cell cycle phases, cohesion and DIC differ in two important aspects. First, acetylation of Smc3 is not sufficient to generate cohesion during DIC, as opposed to S phase cohesion [41,42]. Instead, DNA damage induces Mec1, a DNA damage signaling kinase, to phosphorylate Mcd1 at S83 [41]. Phosphorylated Mcd1 recruits Eco1 which in turn acetylates Mcd1 at K84 and K210, resulting in DIC [42]. Loss of Mcd1 acetylation abolishes DIC and inhibits homologous recombination (HR)-based DNA repair, linking defects in DIC to mutagenic forms of DNA repair such as non-homologous end-joining [12,13,39,40,42]. Thus, mutation of *ESCO2*, or defects in pathways through which DIC becomes activated in response to DNA damage, likely play an important role in human development. Second, Eco1 establishes cohesion at both sites of DNA damage and along undamaged chromosomes in response to DSB. Thus, DIC occurs independent of DNA repair fork components, in contrast to S phase cohesion which occurs through recruitment of Eco1 to the DNA replication fork by PCNA [5,12,13,23,31].

Given that Eco1 is degraded during late S, how is DIC established during M phase? Notably, overexpression of Eco1 in M can drive a second round of cohesion in the absence of DNA damage [42]. This suggests that DNA damage may stabilize Eco1 protein to promote DIC during M phase. Based on elegant biochemical studies that document sequential phosphorylation leading to Eco1 ubiquitination and degradation, one plausible model of Eco1 stabilization during M phase is that Eco1 phosphorylation is inhibited, blocking subsequent ubiquitination and degradation [34–36,43–45]. A second mode of Eco1 stabilization might include directly reducing SCF ubiquitination activity and/or proteasome degradation. Here, we test these models. The results reveal that the degradation machinery is fully operational during DIC, but that elevated rates of Eco1 transcription outpace Eco1 degradation. We further report that this transcription relies in part on Yap5—a transcription factor currently implicated in stress responses [46–48]. Our results revise current models of Eco1 regulation.

## Materials & methods

### Yeast strains

*Saccharomyces cerevisiae* strains used in this study are listed in Table 1.

**Table 1:** 

Strain	Genotype	Reference
YBS1334	*MATa; ECO1-3v5*:*HIS3; A364A*	This study
YMG49	*MATa; lys2*::*KAN; BY4741*	*Saccharomyces* Genome Deletion Project
YMG50	*MATa; yap5*::*KAN; BY4741*	*Saccharomyces* Genome Deletion Project

### General methods

Log phase cultures were maintained at 23°C and normalized to an OD_600_ = 0.2–0.4 in all experiments. G_1_ and S phase arrests in cell cycle time course experiments were achieved by incubating cells in YPD supplemented with Alpha Factor and hydroxyurea (HU; 200mM final concentration) for 3 hours at 23°C, respectively. Alpha Factor and HU was washed out and the cells rinsed with YPD followed by incubation in fresh YPD supplemented with nocodazole (20^μg^/_mL_) at 23°C for the indicated time periods. Assessment of DNA content by flow cytometry were performed as previously described [26]. Translation inhibition was achieved by addition of 100^μg^/_mL_ cycloheximide (Sigma) at 23°C for the indicated time points. DNA damage induction was achieved by addition of 0.1% MMS (Sigma); 333^μg^/_mL_ Zeocin (Invitrogen) or 200mM HU (Sigma) for the indicated time periods at 23°C.

### Western blot analysis

Whole cell extracts were made by TCA precipitation as previously described [49]. Proteins were resolved by SDS PAGE followed by transfer to PVDF membrane using the Trans-Blot Turbo Transfer System (Bio-Rad). Western blot analysis for Eco1 was performed using mouse-anti-V5 primary antibody (Invitrogen), Goat-anti-mouse HRP secondary (Bio-Rad) followed by detection with ECL Prime (GE). To assess Rad53 phosphorylation, samples of MMS-treated cells were prepared by TCA precipitation in the presence of 1% PPI3 phosphatase inhibitor (Sigma). Membranes were probed with anti-Rad53 primary antibody (Santa Cruz, yC-19 for Fig 2C; Abcam, ab104232 for Fig 2G and S2C Fig) and Donkey-anti-Goat HRP/Goat-anti-Rabbit HRP secondary antibody. All images were obtained by exposing ECL treated blots onto X-ray film followed by scanning with an office scanner (EPSON Perfection V300 Photo). Western blot quantification analysis is shown to represent variation between biological replicates and was performed using ImageJ by normalizing Eco1 signal to the loading control PGK within respective lanes. Normally distributed peaks were measured using the entire area of the signal. Quantifications represent one measurement of each sample. Histograms represent quantification of the averaged amount of normalized Eco1 protein within each drug-treated sample divided by the normalized Eco1 protein level in the untreated sample.

### RNA extraction and quantitative real-time PCR

Cells were pelleted by centrifugation and immediately frozen in liquid nitrogen. RNA extraction was performed using the RNeasy Mini Kit (Qiagen) per the manufacturer’s instructions. Cell lysis was performed by bead beating in buffer RLT supplemented with 10μl BME/mL. Purification of total RNA was followed by subsequent RNA clean-up using the RNeasy mini kit (Qiagen) per the manufacturer’s instructions. Quantitative Real-Time (qRT) PCR was performed using the QuantiNova SYBR Green RT-PCR kit (Qiagen) and C_T_ values measured using the Rotor-gene (Corbett). The C_T_ values for *ECO1* and the internal control, the 26S proteasome subunit, *RPN2*, were averaged. The ΔC_T_ values represent the expression levels of *ECO1* normalized to *RPN2* values, an established internal control for qRT-PCR [50]. ΔΔC_T_ values represent the relative level of gene expression. The fold difference was determined using the ΔΔC_T_ method (2^-ΔΔC^_T_) as previously described [51]. Briefly, the ΔC_T_ is determined by subtracting the average *RPN2* C_T_ from the average *ECO1* C_T_. The standard deviation of the ΔC_T_ is calculated from the standard deviations of the *ECO1* and *RPN2* values using the Comparative Method [52].

## Results

### Eco1 is degraded following DNA replication

**Fig 1:**
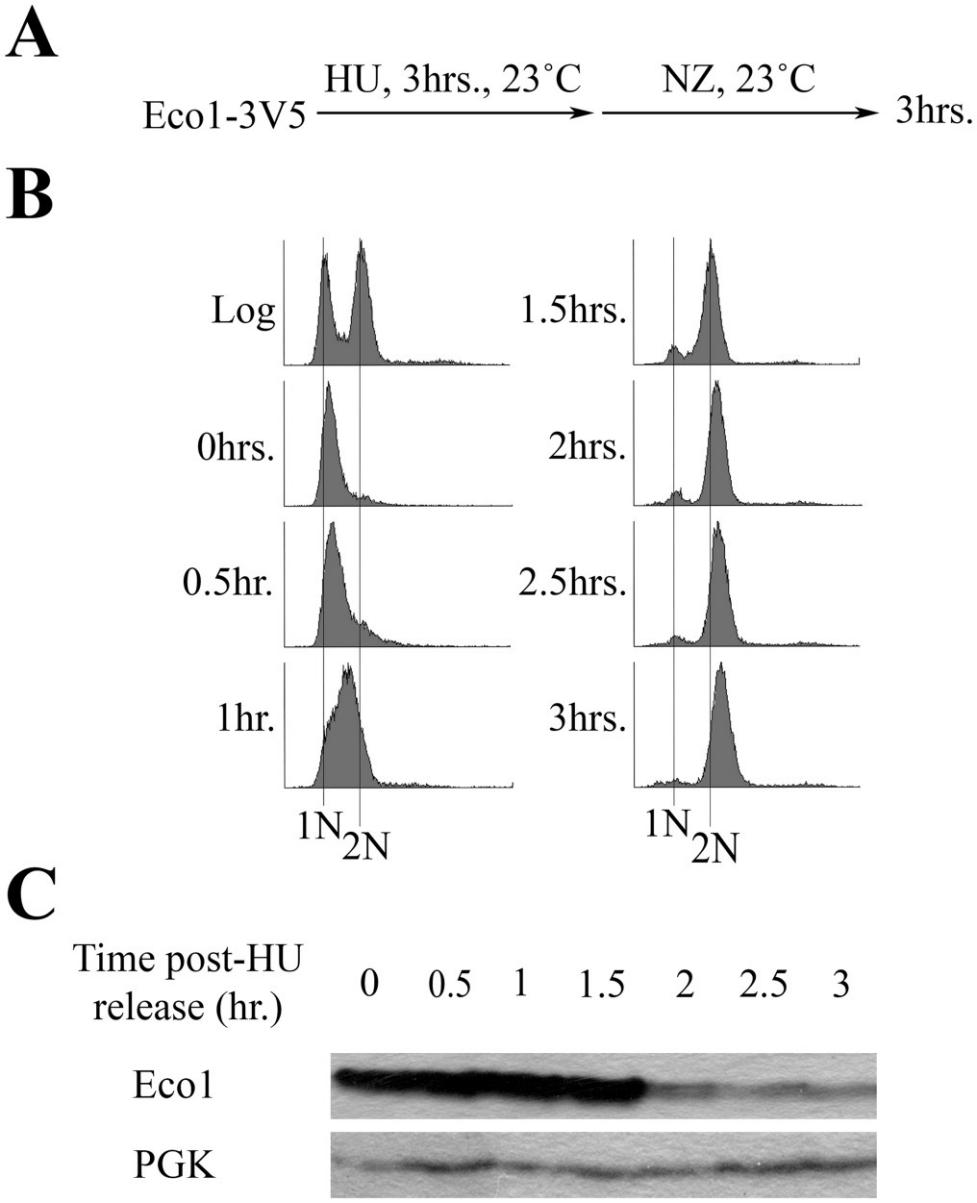
Eco1 protein levels decline after S phase due to targeted degradation. A) Schematic of experimental procedure use to synchronize Eco1-3V5 cells. B) DNA content of cells showcasing cell cycle progression and synchronizations as outlined in (A). C) Detection of Eco1 protein levels (using anti-V5) by Western blot for the time course in (A). PGK detection is used as a loading control.

Eco1-dependent establishment of sister chromatid cohesion is typically limited to S phase [5,6,9,10,24]. This model is supported by cell cycle mapping studies and compelling findings that Eco1 undergoes highly orchestrated and sequential phosphorylation modifications that target Eco1 for degradation during late S phase and through M phase [5,34–36]. Challenging this model, however, are other studies that failed to detect significant changes in Eco1 protein levels across the cell cycle [6,23]. Thus, it became important to independently assess whether Eco1 protein levels change across the cell cycle. Briefly, cells harboring Eco1-3V5, as the sole source of Eco1 protein, were synchronized in early S phase with hydroxyurea (HU), washed and then incubated for three hours in fresh media supplemented with nocodazole (NZ) to achieve an M phase arrest (Fig 1A). Cell cycle progression and Eco1 protein levels were monitored by flow cytometry and Western blot, respectively (Fig 1B and 1C). The results show that Eco1 levels are high during S phase (when cohesion is established) and then drop dramatically as cells progress into M phase (Fig 1C). To determine if Eco1protein level reduction following HU-arrest was an artifact of stalled replication forks, cells harboring Eco1-3V5 were arrested in Alpha Factor for three hours, washed and then incubated for three hours in fresh media supplemented with NZ (S1A Fig). Cell cycle progression and Eco1 protein levels were monitored every hour following Alpha Factor release by flow cytometry and western blot, respectively (S1B and S1C Fig). The results indicate that Eco1 levels are low in G1, peak during DNA replication (S-phase) and decline in M-phase. These results, and those obtained using HU synchronization, are consistent with cell cycle mapping [5] and biochemical studies [34,36].

### DNA damage during m phase induces *de novo* synthesis of Eco1 protein

Eco1 protein levels are very low during M phase, but increase in response to genotoxic agents such as HU, 4-NQ (UV mimetic that induces thymidine dimers) or zeocin (DSB inducer) [34,36]. To independently validate that DNA damage induces an increase in Eco1 protein levels during M phase, both log phase and NZ-arrested cells were treated with 0.1% methyl methanesulfonate (MMS—a DNA alkylating agent) for 1 hr (S2A Fig). Cell cycle state and Eco1 protein levels were monitored by flow cytometry and western blot, respectively (S2B & S2C Fig). In log phase and NZ-arrested cultures, MMS-induced 1.5- and 2.8-fold increases in Eco1 protein levels, respectively, compared to cells not exposed to MMS (S2C Fig). The increase in Eco1 protein levels in both log phase and NZ-arrested cultures coincided with Rad53 phosphorylation (S2C Fig), confirming induction of the DNA damage checkpoint in response to MMS.

**Fig 2:**
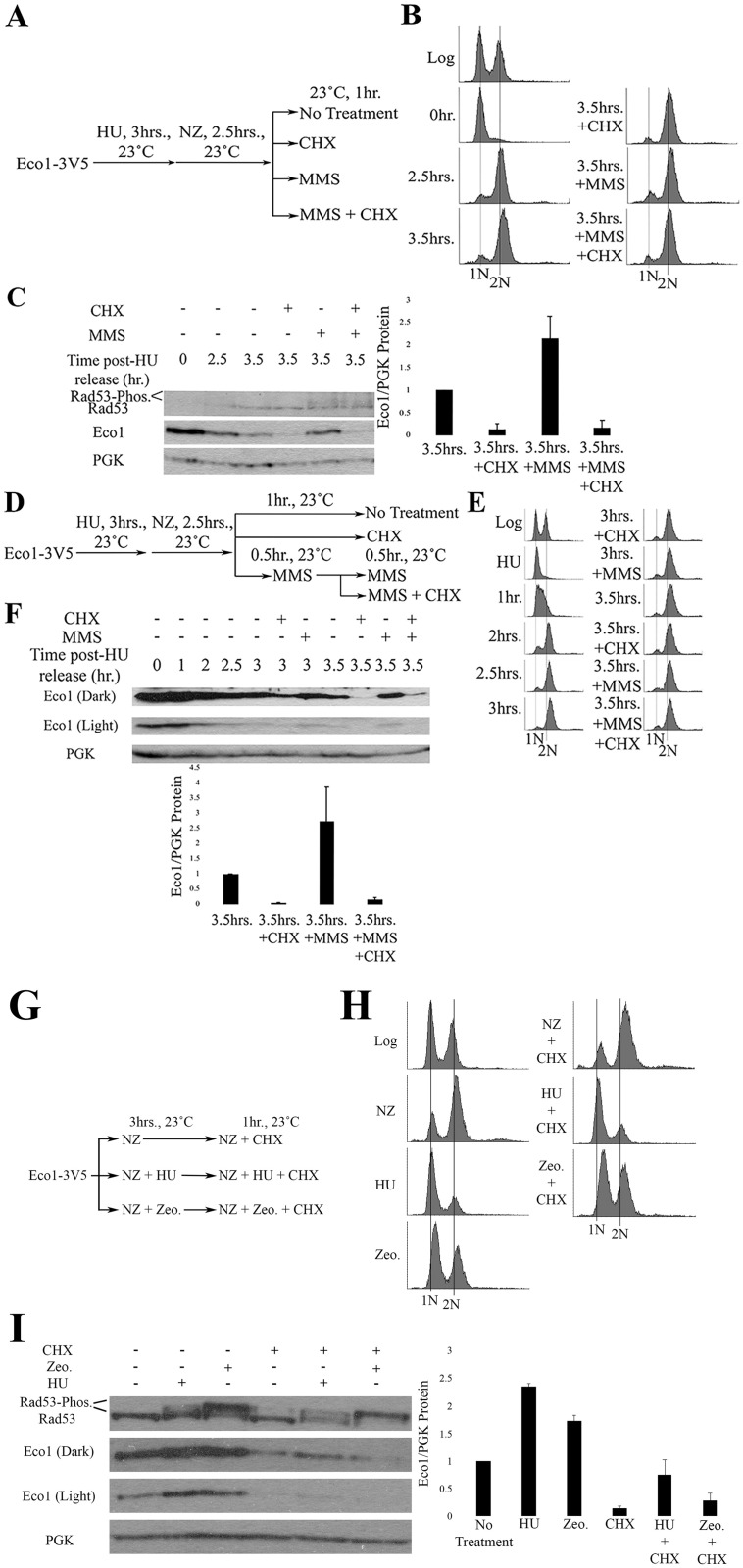
DNA damage induces synthesis of new Eco1 protein. A) Schematic of experimental procedure used to synchronize Eco1-3V5 cells and induce DNA damage during M phase. B) DNA content of Eco1-V5 cells showcasing cell cycle progression and synchronizations. C) Representative western blots (anti-V5) and quantification of Eco1 protein levels, normalized to PGK. Rad53 phosphorylation is provided as a positive control for the induction of DNA damage during M phase. For Eco1 quantifications, N = 3 for all samples at the 3.5hr timepoint. Error bars indicate standard error of the mean. Statistical analysis was performed using a One-tail Student’s T-test. p = 0.037 for the 3.5hr samples, compared to 3.5hr+MMS samples. Statistical differences are based on p<0.1. D) Schematic of experimental procedure used to synchronize Eco1-3V5 cells and induce damage during M phase, by pre-treating cells with MMS, followed by CHX treatment. E) DNA content of Eco1-V5 cells showcasing cell cycle progression and synchronizations. F) Eco1-V5 detection, by Western blot (anti-V5) and quantification for the time course shown in (D). For Eco1-V5 quantification, N = 3 for all samples at the 3.5hr timepoint. Error bars indicate standard error of the mean. Statistical analysis was performed using a One-tail Student’s T-test. p = 0.099 for the 3.5hr, compared to 3.5hr+MMS samples. Statistical differences are based on p<0.1. G) Schematic of experimental procedure used to synchronize Eco1-3V5 cells and induce DNA damage. H) DNA content of Eco1-3V5 cells showcasing cell cycle state and synchronizations. I) Representative western blots and quantification of Eco1 protein levels, normalized to PGK. Rad53 phosphorylation is provided as a positive control for the induction of DNA damage. For Eco1 quantifications, N = 2 for HU and Zeocin samples. N = 3 for NZ + CHX, HU + CHX and Zeocin + CHX samples. Errors bars indicate standard error of the mean. For induction of Eco1 in response to DNA damage, statistical analysis was performed using a One-tail Student’s T-test. p = 0.028 and 0.087 for induction of Eco1 in response to HU and Zeocin compared to untreated (NZ), respectively. Statistical differences are based on p<0.1. For changes in Eco1 protein level in response to DNA damage and CHX treatment, statistical analysis was performed using a Two-tail Student’s T-test. p = 0.045 and 0.143 for HU + CHX and Zeocin + CHX compared to untreated (CHX). Statistical differences are based on p<0.1. p = 0.066 for HU + CHX compared to Zeocin + CHX. Statistical differences are based on p<0.1.

Possible mechanisms for increased Eco1 protein include inhibiting either Eco1 phosphorylation (making Eco1 refractile to SCF-dependent degradation) or the SCF degradation machinery [34,36]. If either of these stabilization-based models is correct, then the rise of Eco1 protein levels that occur in response to DNA damage should persist in cells treated with the translation inhibitor cycloheximide (CHX). To test this prediction, log phase cells were synchronized in early S phase (HU), washed and then released into media supplemented with NZ (Fig 2A). The resulting pre-anaphase cells were then exposed, for 1hr, to either CHX, MMS or both MMS and CHX (Fig 2A). Log phase cell synchronizations in S and M phases were monitored by flow cytometry (Fig 2B). Pre-anaphase cells exposed to MMS for 1hr exhibited a two-fold increase in Eco1 protein levels, compared to cells not exposed to MMS (Fig 2C). Importantly, Eco1 protein levels were instead dramatically reduced in cells co-incubated with both MMS and CHX, compared to cells exposed to MMS alone (Fig 2C). Rad53 phosphorylation was detected in cells treated with MMS alone and also cells co-treated with MMS and CHX (Fig 2), indicating the efficacy of DNA damage induction in both treatments. These results challenge the prediction stated above and suggest instead that the degradation machinery is active and targeting Eco1 during M phase, even in the presence of DNA damage.

Given that Eco1 protein levels fail to increase in M phase cells co-incubated in MMS and CHX, we were concerned that CHX may deplete Eco1 protein levels before the DNA damage response inhibits Eco1 degradation. The fact that Eco1 protein levels remained low during CHX and MMS co-incubation further suggested that the degradation machinery was fully active, despite the presence of DNA damage. To test both of these models, we increased Eco1 protein (MMS) prior to CHX. Log phase cells were synchronized in early S phase (HU), washed, and released into fresh medium containing NZ to arrest cells in preanaphase (Fig 2D). Cell cycle progression was monitored using flow cytometry (Fig 2E). The resulting M phase cells were divided into three cultures, each of which contain NZ to maintain the pre-anaphase state (Fig 2D). The first culture was untreated for the remainder of the experiment—1 hour. The remaining two cultures were exposed to either CHX for 1 hour or MMS for 30 minutes (the latter to obtain elevated Eco1 protein levels) before subsequent addition of CHX for the remaining 30 minutes (Fig 2D). Replicates in which Eco1 protein levels at the 2.5hr time point were less than the 3.5hr time points, despite MMS addition, were excluded from further analyses. As expected, Eco1 protein levels increased 2.5 fold following 1 hour treatment of MMS (Fig 2F). Even a 30 minute exposure to MMS was sufficient to increase Eco1 protein levels 1.5 fold, compared to untreated samples. Subsequent addition of CHX to MMS-treated cells, however, resulted in a dramatic decrease in Eco1 protein levels (Fig 2F).

Eco1 protein levels remain stable in cells treated with CHX after pre-treatment with HU and Zeocin [34]. This observation raised the possibility that our results could be attributed to a drug-specific effect of MMS treatment. To address this possibility, we incubated log phase cells in NZ, HU + NZ or Zeocin + NZ for three hours followed by subsequent treatment with CHX for one hour (Fig 2G). Cell cycle state and Eco1 protein levels were monitored by flow cytometry and western blot, respectively (Fig 2H & 2I). Exposure to HU and Zeocin resulted in 2.4- and 1.8-fold increases in Eco1 protein levels, relative to cells treated with NZ alone (Fig 2I). Importantly, subsequent treatment with CHX for one hour significantly reduced Eco1 protein levels that were upregulated in response to either HU and Zeocin. Eco1 protein levels remained higher in HU-treated cultures compared to Zeocin-treated cultures (Fig 2I). Rad53 phosphorylation was detected in HU- and Zeocin-treated cells, indicating successful induction of the DNA damage response. In summary, these results reveal that 1) Eco1-targeting and degradation machinery is regulated not by the presence of DNA damage but by replication origin firing and 2) that DNA damage causes Eco1 levels to increase (possibly through a transcription-based mechanism) in a manner that significantly outpaces the rate of Eco1 turnover.

### DNA damage induces Eco1 transcription

**Fig 3:**
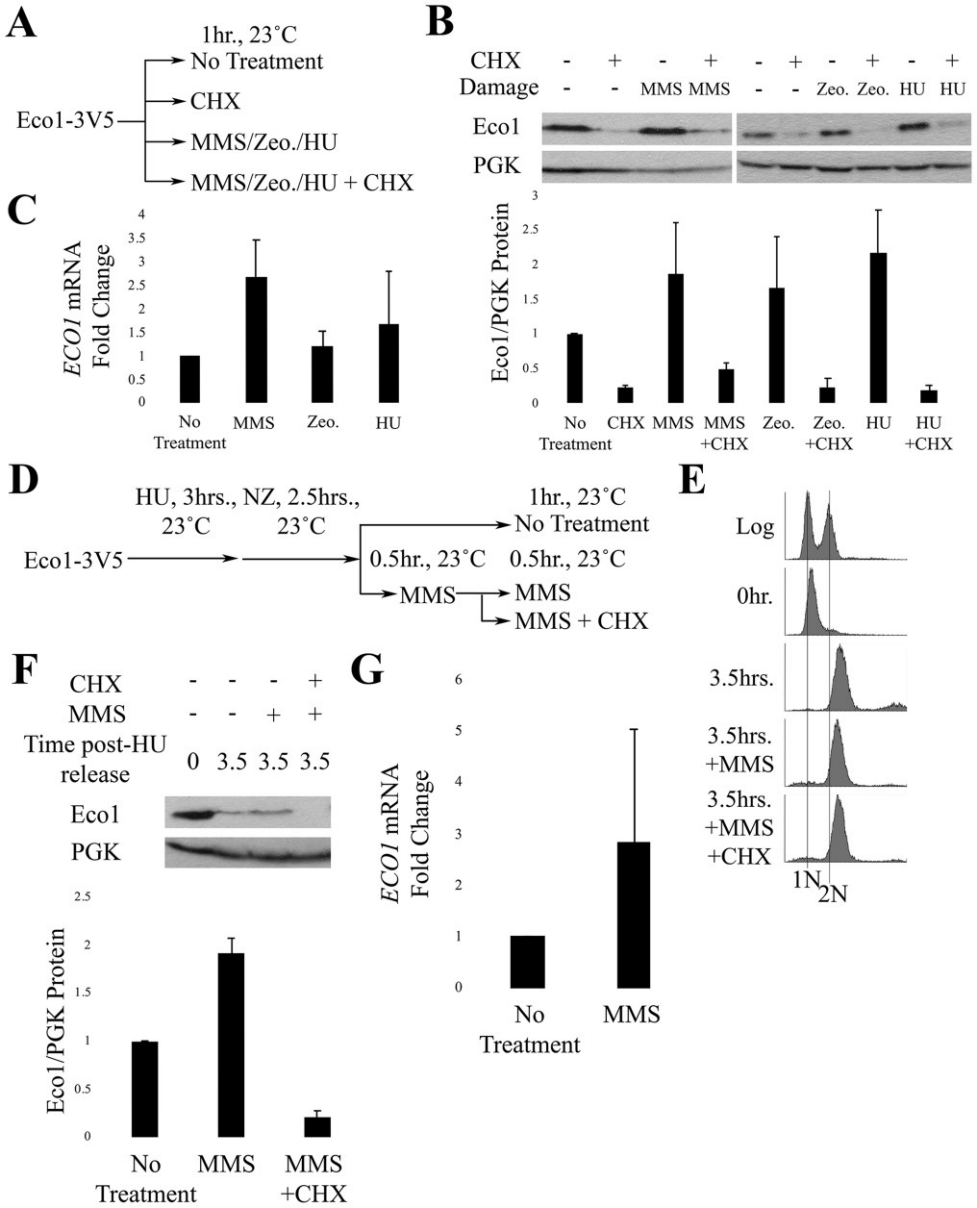
DNA damage induces Eco1 transcription. A) Schematic of experimental procedure. B) Representative western blot and quantification of Eco1-V5 cells for the experiment described in (A). For Eco1-V5 quantification, N = 2 for MMS-, Zeocin- and HU-treated samples. Error bars indicated standard error of the mean. Statistical analysis was performed using a One-tail Student’s T-test. p = 0.117, 0.116, 0.099 for the untreated compared to MMS-, Zeocin- and HU-treated samples, respectively. Statistical differences are based on p<0.1. C) qRT-PCR data from the experiment in (A). Eco1 expression levels are shown for cells treated with MMS (N = 3), Zeocin (N = 3), or HU (N = 2). Error bars indicate standard error of the mean. Statistical analysis was performed using a One-tail Student’s T-test. p = 0.052, 0.273, 0.313 for cells treated with either MMS, Zeocin or HU, respectively, compared to untreated. Statistical differences are based on p<0.1. D) Schematic of experimental procedure used to synchronize Eco1-3V5 cells and perform damage induction in M phase with MMS. E) DNA content of Eco1-V5 cells showcasing cell cycle progression and synchronizations for strategy shown in (D). F) Representative western blot and quantification of Eco1-V5 for the time course in (D). For Eco1-V5 protein level quantifications, N = 2 for all samples at the 3.5hr timepoint. Error bars indicate standard error of the mean. Statistical analysis was performed using a One-tail Student’s T-test. p = 0.016 for the 3.5hr, compared to 3.5hr+MMS samples. Statistical differences are based on p<0.1. G) qRT-PCR data from the experiment shown in (D). Eco1 expression levels of cells treated with MMS only. Error bars indicate standard error of the mean. Statistical analysis was performed using a One-tail Student’s T-test. p = 0.241 for the untreated, compared to 3.5hr+MMS samples. Statistical differences are based on p<0.1.

Given that CHX negates the MMS-dependent increase in Eco1 protein levels, it became important to directly test whether the rate of either *ECO1* transcription or translation increases in response to DNA damage. Mid-log phase cells were exposed for 1 hour to either genotoxic agents (MMS, Zeocin, or HU) alone or in combination with CHX (Fig 3A). Eco1 protein and mRNA levels were then quantified by Western blot and qRT-PCR, respectively. In the absence of CHX, individual treatments to either MMS, Zeocin or HU produced an approx. 2-fold increase in Eco1 protein; a response which trends toward statistical significance (Fig 3B). The addition of CHX, however, abolished induction of Eco1 protein levels in response to each of the DNA damaging agents (Fig 3B). We then tested whether the increase in Eco1 protein corresponded to increased mRNA levels. Results from qRT-PCR revealed a trend indicating increases in *ECO1* mRNA under all conditions of genotoxic stress, although these results were not statistically significant (Fig 3C; S1 Table). Thus, DNA damage increases *ECO1* expression to promote high fidelity DNA repair.

DIC is an M-phase response to DNA damage, prompting us to test whether DNA damage-induced *ECO1* expression occurs specifically during M phase? To address this question, we modified the above experiment to arrest cells in preanaphase following release from an S phase synchronized culture (Fig 3D). The resulting pre-anaphase cells were divided into three cultures, the first untreated for 60 minutes. The second was incubated in medium containing MMS for 60 minutes, while the third was incubated in MMS for 30 minutes followed by co-incubation with MMS and CHX for an additional 30 minutes (Fig 3E). Western blot analyses of whole cell lysates reveal that Eco1 protein levels clearly rise during M phase in response to MMS (Fig 3F), but this increase is completely abolished by subsequent addition of CHX (Fig 3F). Importantly, qRT-PCR analysis revealed trends for increased *ECO1* mRNA levels in M-phase cells exposed to MMS, although these results were not statistically significant (Fig 3G; S2 Table). Taken together, these findings reveal that DNA damage induces *ECO1* transcription, even during M phase, at a level that exceeds that of Eco1 degradation.

### DNA damage-induced transcription of Eco1 is Yap5-dependent

**Fig 4:**
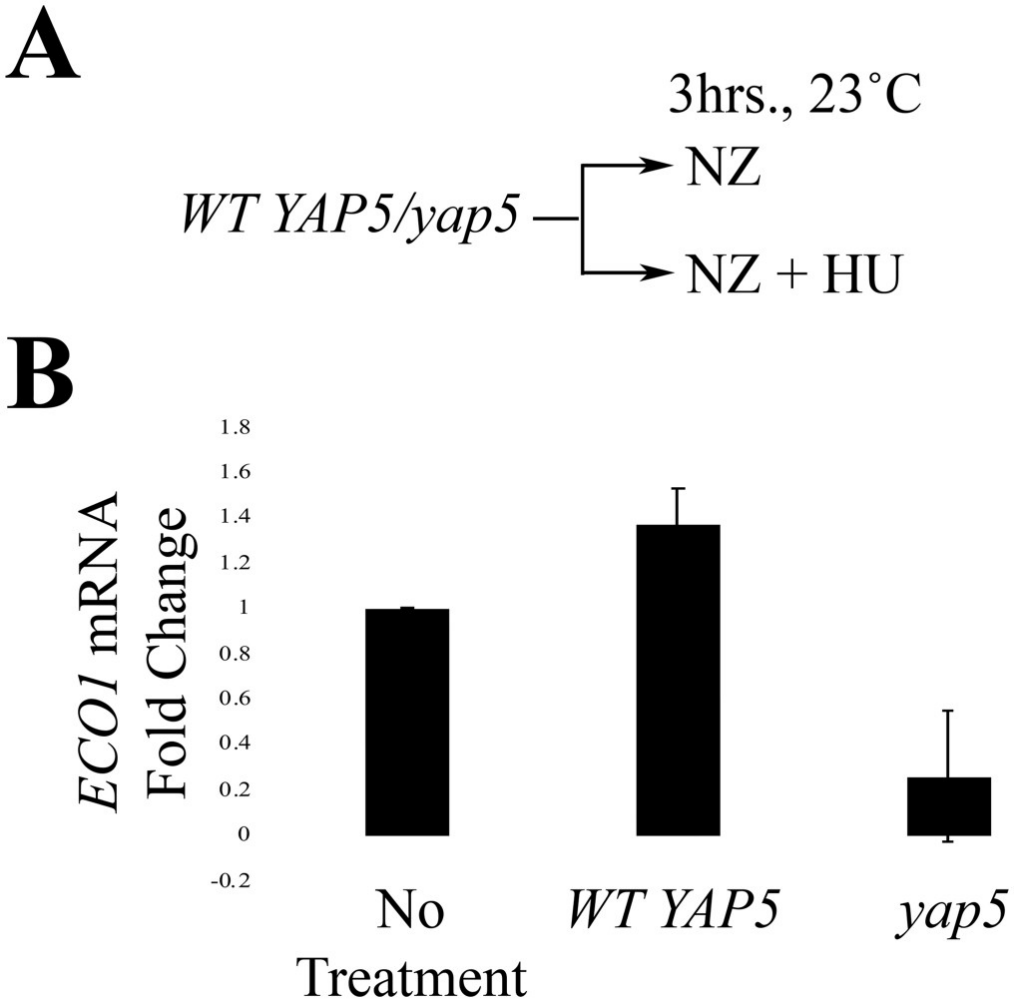
DNA damage induces Yap5-dependent transcription of *ECO1*. A) Schematic of experimental procedure used to synchronize *wildtype* and *yap5* mutant cells and induce DNA damage (HU). B) qRT-PCR data from the experiment strategy shown in (A) (N = 3). Error bars indicate standard error of the mean. p = 0.043 for the untreated, compared to HU-treated samples in the WT strain (One-tail Student’s T-test). p = 0.085 for the untreated vs. HU-treated samples in the *yap5* strain (Two-tail Student’s T-test). p = 0.005 for WT vs. *yap5* (Two-tail Student’s T-test). Statistical differences are based on p<0.1 for all tests.

Yap5 is an iron-sensitive and stress response transcription factor implicated in Eco1 regulation [48]. We hypothesized that the induction of *ECO1* transcription, in response to DNA damage, might occur through a Yap5-dependent mechanism. To test this hypothesis, we exploited a DNA damage induction experiment previously performed by Morgan and colleagues [34] (Fig 4A). Briefly, log phase *WT* and *yap5* cells were treated with either NZ or NZ and HU for 3hrs at 23°C in order to compare *ECO1* mRNA levels within one cell cycle between the two strains (Fig 4A). We also monitored changes in *RNR3* expression as a positive control [53]. As expected, *RNR3* mRNA levels increased dramatically in response to HU (S3 Table). *ECO1* mRNA levels also rose in response to HU (Fig 4B; S4 Table), consistent with our prior results. Importantly, *ECO1* mRNA levels were significantly repressed in *yap5* mutant cells, despite the presence of HU (Fig 4B; S4 Table). Taken together, these results suggest that Yap5 is necessary and sufficient for induction of *ECO1* transcription in response to DNA damage.

## Discussion

**Fig 5:**
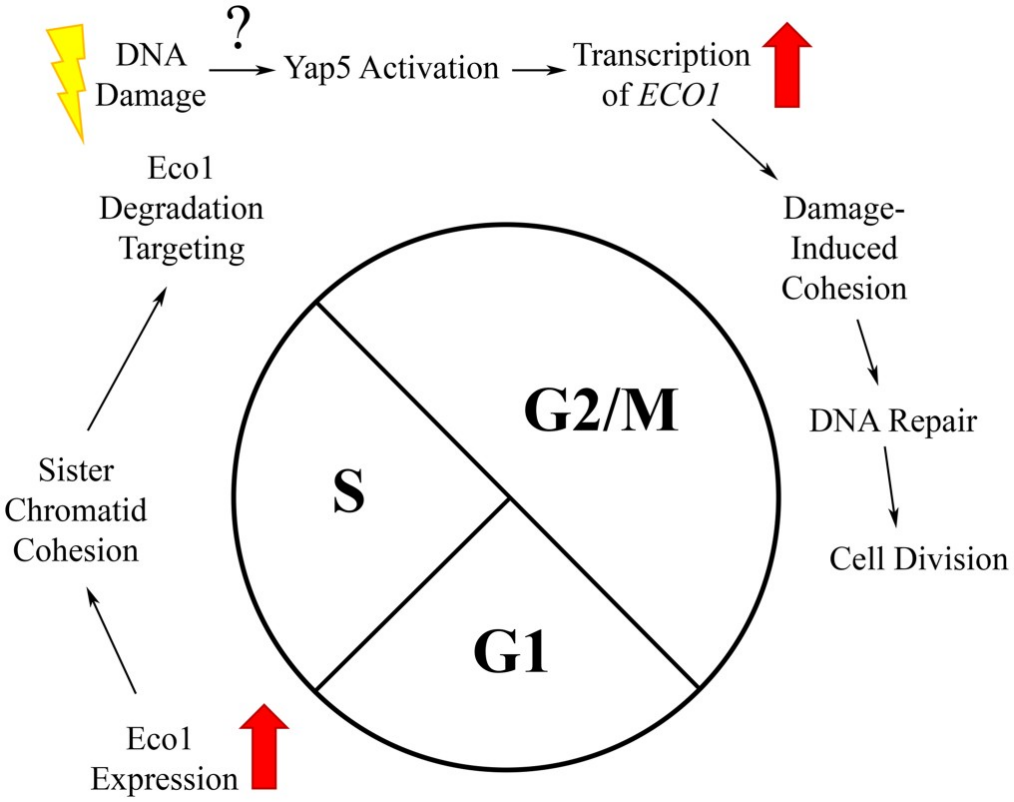
Summary model of Eco1 regulation in response to DNA damage. In unperturbed cells, *ECO1* expression is upregulated at the G1/S transition, enabling sister chromatid cohesion establishment during S phase. Exit from S phase results in Eco1 phosphorylation/ubiquitination and subsequent degradation—which persists through mitosis. In response to DNA damage during M phase, however, Yap5 induces new expression of *ECO1* at a rate that exceeds the rate of Eco1 degradation.

Mutations that occur through either endogenous or exogenous genotoxic agents contribute to both aging and diseases such as cancer. Cohesin is critical for DSB repair in a process that requires the acetyltransferase Eco1 [12,13,39,40,42]. The first major finding of the current study is elucidation of the mechanism through which Eco1 becomes reactivated during M phase and in response to DNA damage (Fig 5). Previous work revealed the central mechanism through which Eco1 activity is limited to S phase: sequential phosphorylations (Cdk1, DDK and Mck1 kinase) that promote ubiquitination (SCF E3 ligase) and subsequent degradation (proteasome) [34,36]. These findings formally raised the possibility that Eco1 reactivation during M might occur either by blocking Eco1 phosphorylation or degradation. Instead, our results reveal that DNA damage results in a significant increase in Eco1 protein levels despite continued Eco1 degradation. This increase is solely due to new synthesis, providing a transcriptional view of Eco1-dependent DNA repair (Fig 5).

How is Eco1 transcription regulated? Early work mapped Eco1 function specifically to S phase and that *ECO1* transcription peaks during S phase [5,54], but a mechanism for *ECO1* induction in response to DNA damage during M phase remained unknown. A second major finding of the current work is that Yap5 is critical for *ECO1* upregulation in response to DNA damage. Our findings extend the role of Yap5, a transcription factor that induces *ECO1* transcription in response to elevated iron levels [48], to include a DNA damage response. Both DNA damage and iron can generate reactive oxygen species (ROS). In turn, ROS triggers an oxidative stress response that protects against macromolecular damage. This high-lights cross-talk between the DNA repair and oxidative stress responses in which Yap5 likely plays a role [55–67]. Other transcription factors in the YAP family, most notably Yap1, have been implicated in both the yeast oxidative stress and DNA damage response pathways [60,63,65,68–72]. Intriguingly, Yap1 and Yap5 bind to the same consensus sequence in DNA and are proposed to overlap functionally [47]. Therefore, control of *ECO1* transcription might represent a unified and conserved pathway that evolved to promote redundancy in protecting the genome from a host of genotoxic agents.

The upregulation of *ECO1*, in response to DNA damage, has implications to human diseases and development. Roberts syndrome is currently modeled as a mitotic failure syndrome [73–77], although emerging evidence supports instead a major role in transcription dysregulation [78–80]. It is important to note in addition that both human and yeast models of RBS exhibit genotoxic sensitivity. We posit that defects in appropriate DNA damage responses, that include a failure to upregulate ESCO2, may constitute a critical but as yet unrecognized role in developmental defects [81–83]. The findings presented here demonstrate the complexity of cellular responses to DNA damage and provide tools for future endeavors that may further link Eco1 function and expression in disease and development.

## Supporting information

S1 Fig Eco1 protein levels are low in G1 phase, peak during S phase and decline in M phase due to targeted degradation. A) Schematic of experimental procedure used to synchronize Eco1-3V5 cells. B) DNA content of cells showcasing cell cycle progression and synchronizations as outlined in (A). C) Detection of Eco1 protein levels (using anti-V5) by Western blot for the time course in (A). PGK detection is used as a loading control.
(TIF)

S2 Fig MMS-induced DNA damage induces Eco1 upregulation in log phase and M phase-arrested cells. A) Schematic of experimental procedure used to synchronize Eco1-3V5 cells and induce DNA damage. B) DNA content of Eco1-3V5 cells showcasing cell cycle progression and synchronizations. C) Representative western blots and quantification of Eco1 protein levels, normalized to PGK. Rad53 phosphorylation is provided as a positive control for the induction of DNA damage. N = 2 for Eco1 quantifications. Errors bars indicate standard error of the mean.
(TIF)

S1 Table Raw qRT-PCR data for Fig 3C. (DOCX)

S2 Table Raw qRT-PCR data for Fig 3G. (DOCX)

S3 Table Raw qRT-PCR data for *RNR3* induction in response to HU. (DOCX)

S4 Table Raw qRT-PCR data for Fig 4B. (DOCX)
