# Supplementary figures and images for: DNA damage induces Yap5-dependent transcription of ECO1/CTF7 in Saccharomyces cerevisiae
Source: PLoS One. 2020 Dec 29;15(12):e0242968. doi: 10.1371/journal.pone.0242968 (PMC7771704; doi:10.1371/journal.pone.0242968)

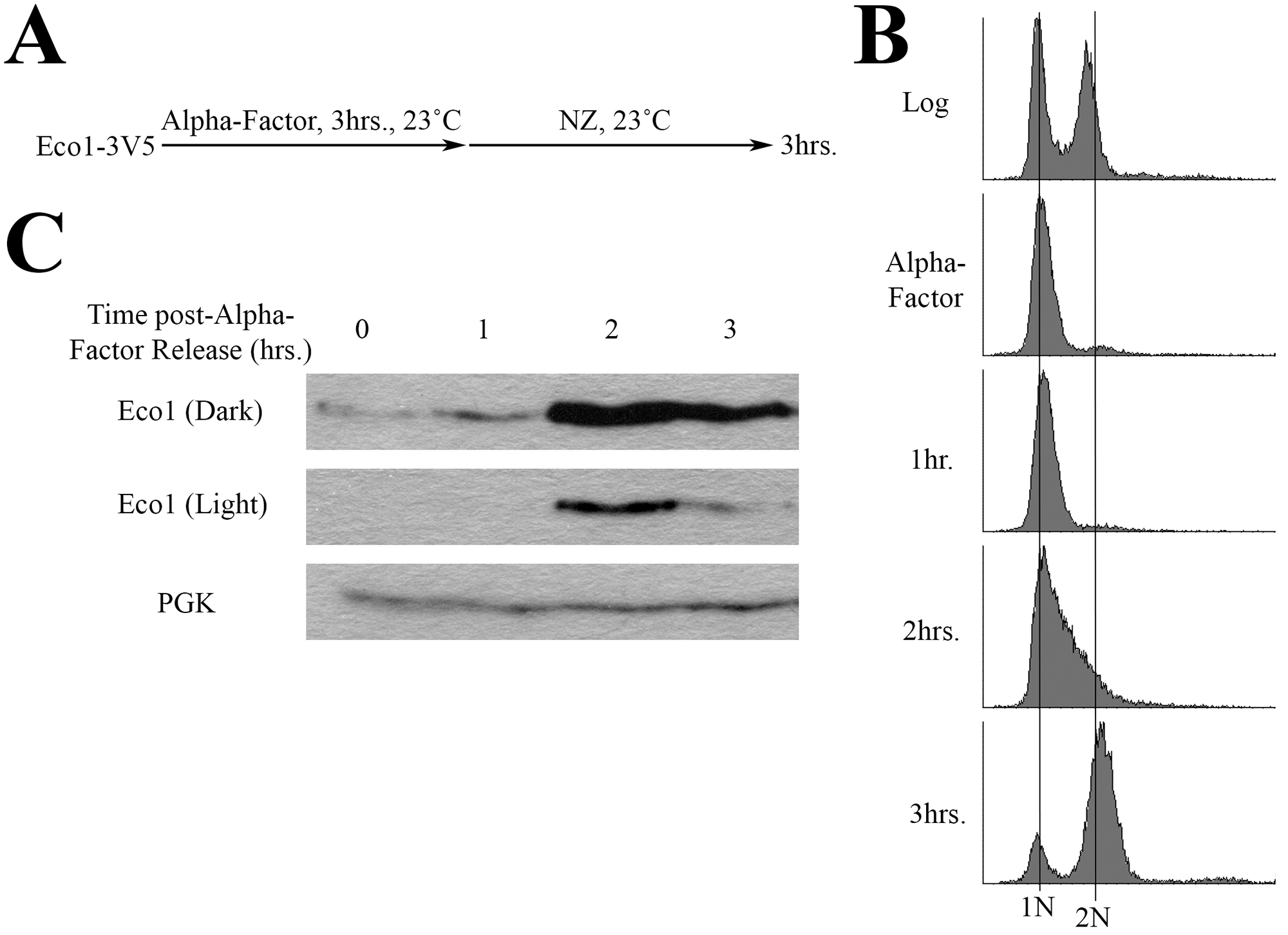

Supplement: S1 Fig — A) Schematic of experimental procedure used to synchronize Eco1-3V5 cells. B) DNA content of cells showcasing cell cycle progression and synchronizations as outlined in (A). C) Detection of Eco1 protein levels (using anti-V5) by Western blot for the time course in (A). PGK detection is used as a loading control. (TIF) [file pone.0242968.s001.tif]

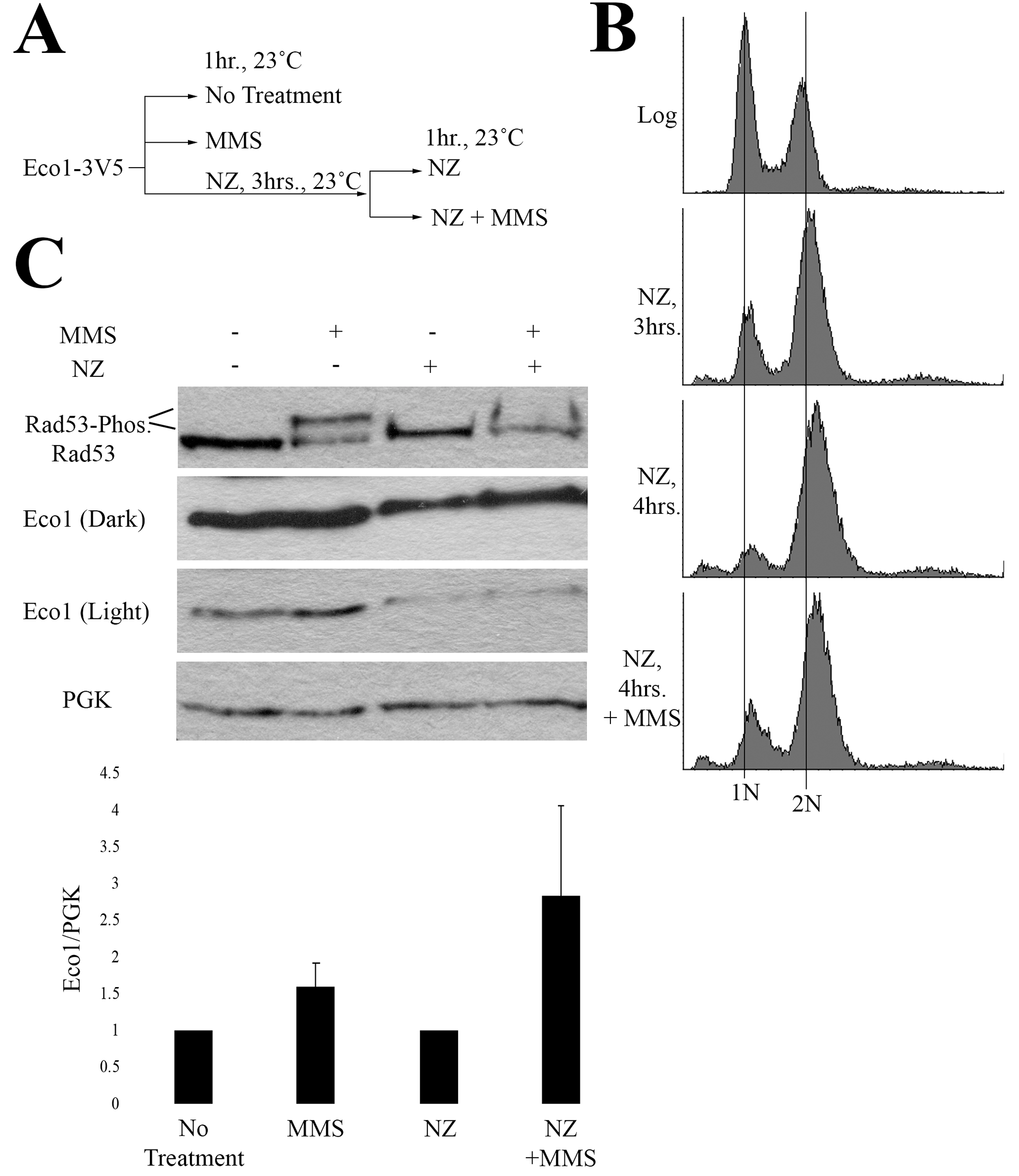

Supplement: S2 Fig — A) Schematic of experimental procedure used to synchronize Eco1-3V5 cells and induce DNA damage. B) DNA content of Eco1-3V5 cells showcasing cell cycle progression and synchronizations. C) Representative western blots and quantification of Eco1 protein levels, normalized to PGK. Rad53 phosphorylation is provided as a positive control for the induction of DNA damage. N = 2 for Eco1 quantifications. Errors bars indicate standard error of the mean. (TIF) [file pone.0242968.s002.tif]
