# Supplementary material for: DNA damage induces Yap5-dependent transcription of ECO1/CTF7 in Saccharomyces cerevisiae
Source: PLoS One. 2020 Dec 29;15(12):e0242968. doi: 10.1371/journal.pone.0242968 (PMC7771704; doi:10.1371/journal.pone.0242968)
Supplement: S1 Table — (DOCX) [file pone.0242968.s003.docx]

**Supplemental Table S1 –** Raw qRT-PCR Data for Figure 3C

| **Treatment** | **Average *ECO1* C_T_** | **Average *RPN2* C_T_** | **△C_T_ *ECO1-RPN2*** | **△△C_T_ (Avg. △C_T_ Exp. - Avg. △C_T_ Con.)** | **Fold Change** |
| --- | --- | --- | --- | --- | --- |
| Untreated | 19.74 +/-  0.68 | 14.8 +/-  1.1 | 4.94 +/-  1.29 | -0.75 +/-  1.92 | 2.68 (0.44-6.36) |
| MMS | 18.36 +/-  1.73 | 14.17 +/-  0.84 | 4.19 +/-  1.92 |  |  |
| Untreated | 18.50 +/-  0.43 | 14.19 +/-  0.11 | 4.31 +/-  0.44 | -0.27 +/-  0.6 | 1.21 (0.80-1.83) |
| Zeocin | 17.75 +/-  0.40 | 13.71 +/-  0.44 | 4.04 +/-  0.60 |  |  |
| Untreated | 18.74 +/-  0.51 | 14.22 +/-  0.12 | 4.52 +/-  0.52 | -0.73 +/-  0.72 | 1.66 (1.01-2.73) |
| HU | 17.77 +/-  0.49 | 13.98 +/-  0.53 | 3.79 +/-  0.72 |  |  |
