# Supplementary material for: DNA damage induces Yap5-dependent transcription of ECO1/CTF7 in Saccharomyces cerevisiae
Source: PLoS One. 2020 Dec 29;15(12):e0242968. doi: 10.1371/journal.pone.0242968 (PMC7771704; doi:10.1371/journal.pone.0242968)
Supplement: S2 Table — (DOCX) [file pone.0242968.s004.docx]

**Supplemental Table S2 –** Raw qRT-PCR Data for Figure 3G

| **Treatment** | **Average *ECO1* C_T_** | **Average *RPN2* C_T_** | **△C_T_ *ECO1-RPN2*** | **△△C_T_ (Avg. △Ct Exp. - Avg. △Ct Con.)** | **Fold Change** |
| --- | --- | --- | --- | --- | --- |
| Untreated | 19.37 +/- 0.98 | 14.32 +/- 0.10 | 5.05 +/- 0.99 | -1.5 +/- 0.35 | 2.83 (2.22-3.61) |
| MMS | 17.41 +/- 0.17 | 13.86 +/- 0.31 | 3.55+/- 0.35 |  |  |
