# Supplementary material for: DNA damage induces Yap5-dependent transcription of ECO1/CTF7 in Saccharomyces cerevisiae
Source: PLoS One. 2020 Dec 29;15(12):e0242968. doi: 10.1371/journal.pone.0242968 (PMC7771704; doi:10.1371/journal.pone.0242968)
Supplement: S3 Table — (DOCX) [file pone.0242968.s005.docx]

**Supplemental Table S3-** Raw qRT-PCR Data for *RNR3* induction in response to HU

| **Strain** | **Treatment** | **Average**  ***RNR3* C_T_** | **Average**  ***RPN2* C_T_** | **△C_T_ *RNR3-RPN2*** | **△△C_T_ (Avg. △C_T_ Exp. - Avg. △C_T_ Con.)** | **Fold Change** |
| --- | --- | --- | --- | --- | --- | --- |
| WT | Untreated | 23.51 +/-  2.46 | 16.19 +/-  3.29 | 7.32 +/-  4.12 | -5.33 +/- 2.85 | 40.22 (5.58-290.02) |
|  | HU | 19.12 +/-  2.53 | 17.13 +/-  1.32 | 1.99 +/-  2.85 |  |  |
| *△Yap5* | Untreated | 23.8 +/-  2.85 | 16.53 +/-  2.43 | 7.27 +/-  3.75 | -4.74 +/- 3.17 | 26.72 (2.97-240.52) |
|  | HU | 19.23 +/-  2.24 | 16.7 +/-  2.24 | 2.53 +/-  3.17 |  |  |
