# Supplementary material for: DNA damage induces Yap5-dependent transcription of ECO1/CTF7 in Saccharomyces cerevisiae
Source: PLoS One. 2020 Dec 29;15(12):e0242968. doi: 10.1371/journal.pone.0242968 (PMC7771704; doi:10.1371/journal.pone.0242968)
Supplement: S4 Table — (DOCX) [file pone.0242968.s006.docx]

**Supplemental Table S4-** Raw qRT-PCR Data for Figure 4B

| **Strain** | **Treatment** | **Average *ECO1* C_T_** | **Average *RPN2* C_T_** | **△C_T_ *ECO1-RPN2*** | **△△C_T_ (Avg. △C_T_ Exp. - Avg. △C_T_ Con.)** | **Fold Change** |
| --- | --- | --- | --- | --- | --- | --- |
| WT | Untreated | 17.36 +/- 0.48 | 14.24 +/- 0.38 | 3.12 +/- 0.61 | -0.45 +/- 0.99 | 1.37 (0.69- 2.71) |
|  | HU | 17.66 +/- 0.46 | 14.99 +/- 0.88 | 2.67 +/- 0.99 |  |  |
| *△Yap5* | Untreated | 17.4 +/- 0.79 | 14.81 +/- 0.57 | 2.59 +/- 0.97 | 1.95 +/- 2.45 | 0.26 (0.47-1.41) |
|  | HU | 19.58 +/- 2.44 | 15.04 +/- 0.22 | 4.54 +/- 2.45 |  |  |
